# Computational-Aided Approach for the Optimization of Microfluidic-Based Nanoparticles Manufacturing Process

**DOI:** 10.1007/s10439-024-03590-1

**Published:** 2024-08-04

**Authors:** Marco Bellotti, Enrica Chiesa, Bice Conti, Ida Genta, Michele Conti, Ferdinando Auricchio, Alessandro Caimi

**Affiliations:** 1 Department of Civil Engineering and Architecture, Università degli Studi di Pavia, Via Ferrata 3, Pavia, Italy; 2 Department of Drug Sciences, Università degli Studi di Pavia, V.le Taramelli 12, Pavia, Italy

**Keywords:** Nanoparticles, Microfluidics, Numerical simulation, Manufacturing

## Abstract

In the last few years, the microfluidic production of nanoparticles (NPs) is becoming a promising alternative to conventional industrial approaches (e.g., nanoprecipitation, salting out, and emulsification-diffusion) thanks to the production efficiency, low variability, and high controllability of the production parameters. Nevertheless, the development of new formulations and the switching of the production process toward microfluidic platforms requires expensive and time-consuming number of experiments for the tuning of the formulation to obtain NPs with specific morphological and functional characteristics. In this work, we developed a computational fluid dynamic pipeline, validated through an ad hoc experimental strategy, to reproduce the mixing between the solvent and anti-solvent (i.e., acetonitrile and TRIS–HCl, respectively). Moreover, beyond the classical variables able to describe the mixing performances of the microfluidic chip, novel variables were described in order to assess the region of the NPs formation and the changing of the amplitude of the precipitation region according to different hydraulic conditions. The numerical approach proved to be able to capture a progressive reduction of the nanoprecipitation region due to an increment of the flow rate ratio; in parallel, through the experimental production, a progressive increment of the NPs size heterogeneity was observed with the same fluid dynamic conditions. Hence, the preliminary comparison between numerical and experimental evidence proved the effectiveness of the computational strategy to optimize the NPs manufacturing process.

**Graphical Abstract:**

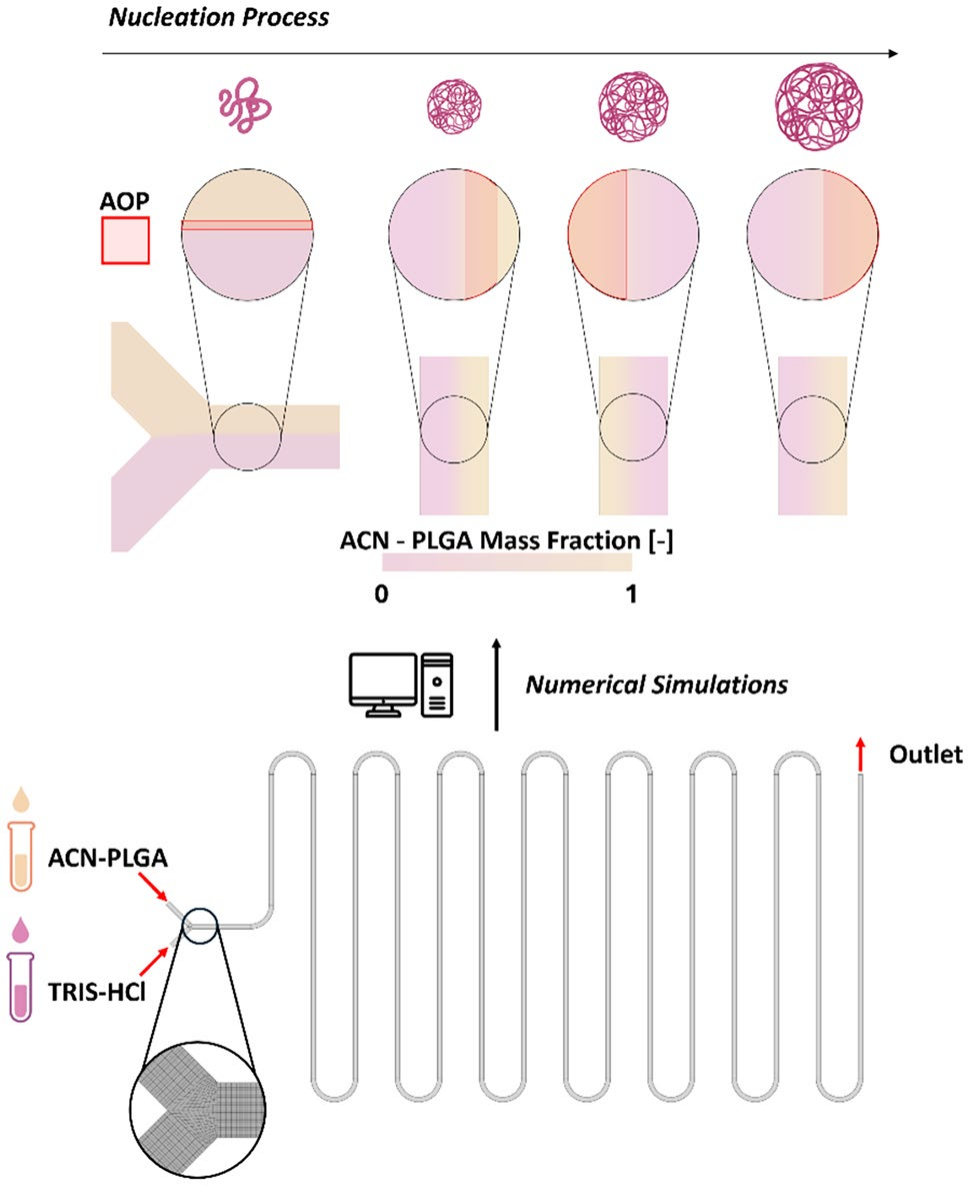

**Supplementary Information:**

The online version contains supplementary material available at 10.1007/s10439-024-03590-1.

## Introduction

Lipid and polymeric Nanoparticles (NPs) are obtained through self-assembly or nanoprecipitation during the mixing of two fluids, a solvent containing an organic precursor (e.g., lipid or polymers) and an aqueous anti-solvent (e.g., buffer solution at different pHs) [1]. Nowadays, polymeric nanoparticles, such as the ones produced in the current study, are commonly used as nanocarrier in gene therapy and in vaccine administration [2].

The manufacturing process is traditionally performed in bulk solution in which the mixing process of the two fluids is controlled by a magnetic stirrer [3]. Nevertheless, the poor batch to batch process reproducibility, the low precision, and the lack of control on the product quality, limit the application of this strategy in an industrial scale [4].

Instead, in the recent years, microfluidics has gained increasing interest for the industrial NPs manufacturing due to many advantages, such as fast processing, low sample volume, excellent control of fluid dynamic variables over the mixing process, and the possibility to rapidly screen the impact of different experimental conditions onto the NPs production [5]. Furthermore, the possibility to control the fluid mixing within an exchangeable microfluidic-based cartridge (i.e., microfluidic chip) allows to maximize the production efficiency limiting as much as possible the volume of waste.

However, the large-scale production process is preceded by an intensive research and development stage in which researchers must explore different working conditions of the microfluidic apparatus in order to develop the optimal formulation for NPs production [6]. To optimize the NPs formulation three different kinds of variables must be accurately regulated: (i) the fluid dynamic working conditions for the liquid-to-liquid interaction (i.e., the total flow rate, TFR, and the flow rate ratio, FRR) [7], (ii) the rheological properties of the different solutions with the addition of the organic precursor [8], and (iii) the geometrical properties of the microfluidic cartridge used for the mixing of the two fluids [9].

According to the pharmaceutical guidelines, in order to investigate the effect of different production variables on the final industrial product, the Design of Experiments (DoE) approach is typically adopted. Moreover, since DoE proved to be an efficient methodology for the pharmaceutical development it is widely used in the industrial world [10]. Nevertheless, since the DoE investigation strategy consists in many different experiments, the R&D cost and time increase tremendously making the production stage very expensive. However, numerical strategies could be helpful in reducing the efforts associated to a complex DoE and therefore they could be effective in formulation’s process optimization.

Numerical strategies, adopting the computational fluid dynamics (CFD) approach, proved to be suitable to assess the mixing characteristics, to optimize novel microfluidic chips [11] and to improve the *quality by design* approach for the development of a different nano-formulation. Nevertheless, most of the works presented in the literature give a wide and exhaustive information about the fluid dynamic performances of the microfluidic cartridges but only few numerical works [12] tried to give preliminary information about the assessment of the NPs precipitation region within the microfluidic chip.

The mixing process was effectively described through different variables (e.g., mixing index (MI) and mixing efficiency (ME)) [13–15], but no variables able to comprehensively assess and predict the NPs production process are present in the literature. Moreover, besides MI, the manufacturing process is deeply related to the residence time and the path experienced by the NPs within the microfluidic cartridge [16].

Hence, starting from a previous experience [9], the authors aim at proposing in silico strategies to assist, support, and simplify the polymeric NPs formulation design and development. In particular, (i) a 0D simplified model was developed to define the limit working conditions admissible by the microfluidic cartridge; (ii) a CFD model was adopted to reproduce the mixing process in the microfluidic chip capturing the effect of hydraulic boundary conditions on the NPs process; (iii) an ad hoc image-based experimental protocol was developed to validate the numerical model; and finally, (iv) different NPs batches were produced and analyzed for a proper correlation of the obtained results with the production evidence. The main advantage of the proposed approach is the reduction of the number of experiments and less consumption of raw materials for the R&D phase notably cutting the cost required for the development of a new nano-formulation.

## Material and Methods

### Reproduction of the Microfluidic Cartridge

#### CAD Geometry and Mesh Reconstruction

**Fig. 1:**
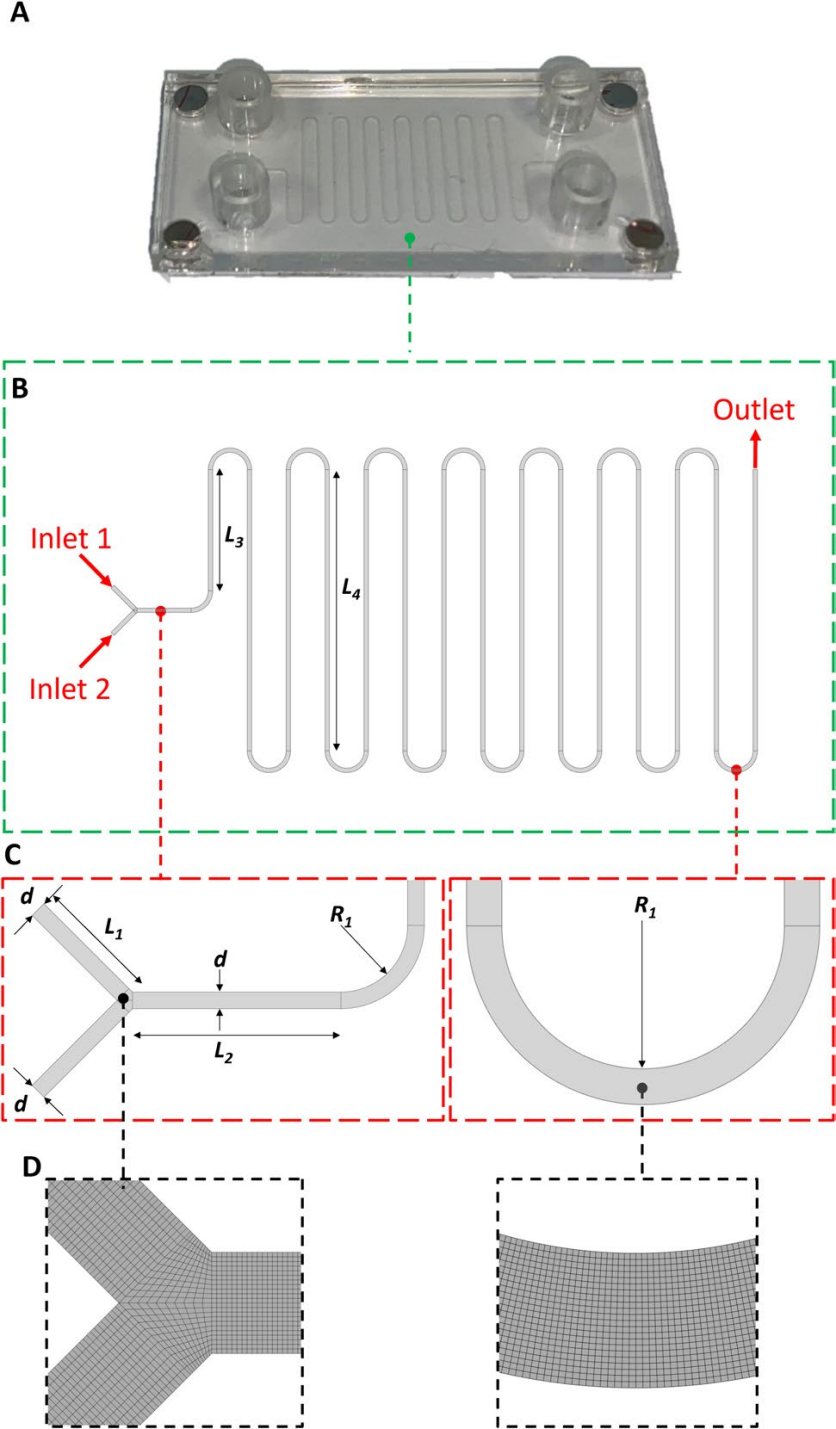
**A** Real image of the Glass Mixing Serpentine Chip. **B** Microfluidic channel reproduced in Ansys Design-Modeler. **C** Magnification of Intersection and Bend, where indicated dimensions refer to Table 1. **D** Meshed domain in correspondence of the intersection and bend.

A glass *serpentine mixing chip* (MesoBioTech, Paris, France) was adopted for the NPs production (Fig. 1A). The chip geometry was reconstructed with ANSYS 2021 R2 (Ansys Inc., Canonsburg, PA, USA) through the *Design-Modeler* dedicated tool following the geometrical features reported in Table 1 and shown in Fig. 1B. In the numerical model a reduced portion of the two inlet channels were considered, the length of the *in silico* inlet channel was chosen ensuring the fully development of the velocity profile before the geometrical junction [17].

**Table 1:** Geometry detail of the modeled microfluidic chip with serpentine structure

L1 (μm)	L2 (μm)	L3 (μm)	L4 (μm)	R1 (μm)	R2 (μm)	d (μm)	h (μm)
1500	2500	5600	13000	800	800	200	100

Moreover, thanks to the strongly laminar flow regime of the microfluidic cartridge (Re ~ 1), thanks to the absence of any secondary flow (Dn <  < 110 [18]), and since the squared microfluidic channel does not present any structure able to alter the flow path, the 3D fluid dynamics of the cartridge can be simplified in a 2D problem-solving fluid dynamic of the central cross-section of the microfluidic chip.

The reconstructed 2D surface was discretized using the *Meshing tool* present in ANSYS 2021 R2 d (Fig. 1D) with a characteristic cell size of 10 μm, chosen after completing a sensitivity analysis. The adopted structured quadrangular grid was characterized by excellent element quality metrics ensuring the goodness of the discretization process and improving the numerical accuracy (i.e., mean Aspect Ratio equal to 1.11, an average Skewness of 7.35e e^−4^, and a mean orthogonal quality of 0.99).

#### 0D Microfluidic Cartridge Simplification

**Fig. 2:**
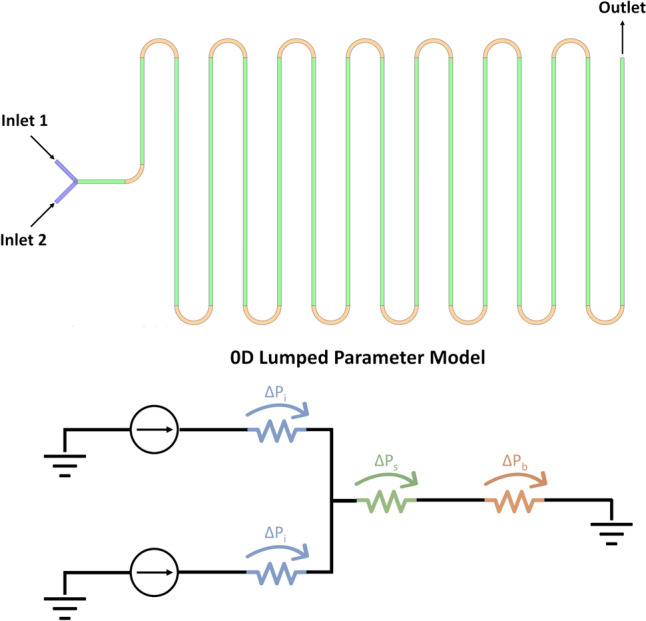
Representation of the Lumped Parameter model of the Mixing Serpentine Chip. In the realistic model (On top) inlet, straight and bended channels are colored in blue, green, and orange, respectively. The effect of these channels can be simplified with hydraulic resistance, in the 0D model, the $\Delta {p}_{i}$ represents the pressure drop induced by each inlet channel, and $\Delta {p}_{s}$ and $\Delta {p}_{b}$, respectively, represent the pressure drop caused by all the straight and bended portion of the channel. The ground symbol represents an outlet null pressure boundary condition.

To determine the flow conditions required to reach the cartridge burst pressure (*i.e.,* 800 mbar) a simplified 0D lumped parameter model was developed (Fig. 2). Since the cartridge is composed of a sequence of straight and curved sections, two different pressure drop definitions were adopted for the model. The pressure drop ($\Delta {p}_{s}$) relative to the straight portions of the chip was calculated adopting the following Hagen–Poiseuille equation through the following relation:

1 $$\Delta {p}_{s}=\frac{32\mu Lv}{{D}_{h}^{2}}$$

where µ represents the dynamic viscosity of the liquid (in this case water, *i.e.*, 1cP), L is the length of the straight portion, *v* is the mean velocity of the fluid, and *D*_*h*_ is the hydraulic diameter of the tube.

The pressure drop relative to the bended channel ($\Delta {p}_{b}$) is calculated through the following relation:

2 $$\Delta {p}_{b}=\frac{K\rho {v}^{2}}{2}=\frac{16R\left(0.026{D}_{n}{e}^{0.661}+1\right)\rho {v}^{2}\theta }{{D}_{h}Re}$$

where $K$ is the bend loss coefficient and it is defined through the approximation of the diagram of Ghia et al. for low Re [19], ρ represents the density of the fluid, *R* is the radius of the bend, *Re* is the Reynolds number, *D*_*n*_ is the Dean number, and θ is the bending angle (*i.e.,* 90° and 180° in this case). Furthermore, since the chip is made of glass and the fluid flowing in the device is characterized by a very low velocity, any compliance, and inertance terms were neglected; finally, for sake of simplicity, the concentrated resistance induced by the connection of the two inlets was not considered. Once the hydraulic characteristics of each part of the microfluidic chip is defined, the limit TFR was easily identified knowing the maximum overall pressure drop acting on the cartridge (*i.e.,* the chip burst pressure).

In order to assess the reliability of the simplified model, different pressure regimes calculated adopting the reduced 0D model were compared with the ones evaluated through a set of CFD simulations (the details of the simulations are reported in the supplementary material).

### Rheological Properties of the Fluid

Among the polymeric materials usually adopted for the NPs production, the poly(lactic-co-glycolic acid) (PLGA) offers several advantages, since it is biocompatible and biodegradable with a tunable degradation rate [20]. The production of PLGA NPs is based on the mixing of a solvent, acetonitrile (ACN), which contains PLGA molecules with a concentration of 7 mg/mL, with an aqueous buffer (*i.e.,* Tris(hydroxymethyl)methylamine, TRIS) [9]. Finally, in order to comprehensively characterize the CFD model, the rheological properties of ACN, TRIS, and the ACN-PLGA solution were assessed through the Kinexus Plus Rheometer (Malvern Panalytical, Malvern UK) testing the fluid with a shear rate range between 0 and 100 ${s}^{-1}$ with a plate–plate system of 60 mm (upper plate diameter) with a gap of 1 mm [21].

The average dynamic viscosity of each solution, reported in Table2, was calculated as the slope of the best linear regression curve able to fit the experimental data obtained by the machine (i.e., shear rate and shear stress); the fitting correlation coefficient (i.e., R^2^) is reported in Table 2 as well.

**Table 2:** Rheological and diffusivity properties of the considered solvents

	Density^a^ (kg/m^3^)	Viscosity (Pa s)	Linear fitting (R^2^) (–)	Diffusion Coefficient^b^ (m^2^/s)
TRIS	1000	0.001	0.99	-
ACN	790	0.0004	0.98	3.5 e^−9^_c_
ACN-PLGA	797	0.0005	0.99	3.5 e^−9^

^a^Calculated as (mass of the solvent + mass of the solute) / volume of the solution.

^b^The considered coefficients, obtained at 298 K, represent the diffusion of the solvent into ant-solvent neglecting PLGA molecular movement.

^c^Solvent diffusion in aqueous buffer as reported in [22].

Moreover, since the NPs production is deeply related to the diffusion of the two solutions into each other, the diffusion coefficients of each solvent in an aqueous buffer was obtained from literature, Table 2.

### Numerical Simulations

The numerical simulations have been performed with ANSYS Fluent 2021 R2 (Ansys Inc., Canonsburg, PA, USA) on a workstation with a quad-core Intel(R) Xeon(R) W-2123 CPU @ 3.60 GHz, 32 GB of RAM, and a NVIDIA Quadro P4000 GPU.

The mixing process was modeled adopting the Species Transport model implemented in ANSYS Fluent 2021 R2 that allows to simulate the mixing of two fluids considering advection and diffusion processes. Since for all the simulated conditions a low Reynolds number was calculated, a *Laminar Viscous* model was adopted.

All the simulations were conducted adopting the ANSYS Fluent steady-state solver with the pseudo transient formulation to guarantee a fast convergence of the result. The *Coupled* algorithm was used for the velocity–pressure coupling with the flux equations resolved with the Rhie–Chow distance-based method. The *PRESTO!* interpolation scheme was used for the pressure calculation while the second order upwind scheme was exploited for the discretization of the Momentum and Diffusion equations. The residual criteria of convergence were set at 10 ${e}^{-6}$ to ensure minimal variation between each iteration.

Thanks to the above-mentioned numerical assumptions, simplified version of *Continuity*, *Navier–Stokes,* and *Species Transport* equations, adopted to solve the proposed problem, are herein reported. The Continuity equation for incompressible fluid can be expressed as function of the fluid density ($\rho$) and velocity (${\varvec{v}}$):

3 $$\nabla \cdot \left(\rho {\varvec{v}}\right)=0$$

Furthermore, since in microfluidics the volumetric forces can be neglected, the Navier–Stokes equation can be expressed in the current form (Eq. 4):

4 $$\nabla \text{p}=\mu \left({\nabla }^{2}{\varvec{v}}\right)$$

Showing the direct dependencies between the pressure change ($\nabla \text{p}$) with the velocity and the viscosity ($\mu )$.

Finally, Eq. 5 represents the species transport equation adopted to solve the mass conservation of different chemical species (${\text{Y}}_{\text{i}}$) characterized by a diffusion coefficient (${\text{D}}_{\text{i}}$) within a control volume.

5 $$\nabla \cdot \left(\uprho \mathbf{v}{\text{Y}}_{\text{i}}\right)+\uprho {\text{D}}_{\text{i}}{\nabla }^{2}{\text{Y}}_{\text{i}}=0$$

The detailed description of the simplification process is reported in the supplementary materials.

#### Boundary Conditions

Four different numerical conditions were adopted to study the NPs formation process. For all the simulations, the same TFR, extracted from the simplified 0D-Model, of 0.2 ml/min was considered, while different FRRs between ACN-PLGA and TRIS were adopted. As a starting point, a FRR of 1 fraction of ACN-PLGA and 1 fraction of TRIS was used, subsequently the ratio was changed increasing the TRIS fraction (i.e., 1:3, 1:5, 1:7).

Different Dirichlet boundary conditions were applied to the model: a no slip condition was prescribed at the wall of the chip and a zero-pressure was superimposed at the cartridge outlet, while different velocity conditions (Table3), according to the TFR and FRR, were set at the two inlets.

**Table 3:** Velocity input boundary conditions

	v_Inlet1_ (m/s)	v_Inlet2_ (m/s)
FRR 1:1	8.33 e^−2^	8.33 e^−2^
FRR 1:3	4.17 e^−2^	1.25 e^−1^
FRR 1:5	3.33 e^−2^	1.33 e^−1^
FRR 1:7	2.08 e^−2^	1.46 e^−1^

The same boundary conditions were used to experimentally produce PLGA NPs; size and PDI (i.e., polydispersity index, an index for the assessment of the homogeneity of population) of the produced NPs were measured adopting a NICOMP 380 ZLS apparatus (Particle Sizing System, Menlo Park, CA, USA) with a 632.8-nm laser and a detection angle of 90° and therefore determining the effect of FRR variation with the serpentine geometry.

### Post-processing

From the results of 2D simulations, the velocity and the mass fraction profiles were extrapolated at different fluid dynamic conditions and elaborated to extract mixing-related parameters. Starting after the inlet Y junction, the mass fraction distribution was extracted at the mid-point of each straight portion of the serpentine chip, for a total of 15 cross-sections. Through the distribution of the mass fraction along the cartridge the mixing efficiency related variable can be assessed.

The Mixing Index [23] is one of the most adopted variables for the assessment of the evolution and the goodness of the mixing profile along the microfluidic channel. In the proposed work, for each cross-section of the channel, the MI was calculated (Eq. 6) as the square root of the ratio between the variance of the mass fraction with respect to the j^th^ cross-section ($\frac{1}{N}{\sum }_{i=1}^{N}{\left({X}_{i}-\overline{{X }_{j}}\right)}^{2})$, and the variance of the mass fraction with respect to the unmixed reference plane $(\frac{1}{N}{\sum }_{i=1}^{N}{\left({X}_{i}-{\overline{X} }_{1}\right)}^{2}$):

6 $${MI}_{j}=\left(1-\sqrt{\frac{\frac{1}{N}{\sum }_{i=1}^{N}{\left({X}_{i}-\overline{{X }_{j}}\right)}^{2}}{\frac{1}{N}{\sum }_{i=1}^{N}{\left({X}_{i}-{\overline{X} }_{1}\right)}^{2}} }\right)\times 100 \quad j=2,\dots 15$$

Concerning the choice of the unmixed reference, the mid-section of the first straight (horizontal) portion of the chip was adopted as representative of the unmixed state.

In the chemistry context [9], the mixing efficiency (ME) is used to evaluate the presence of unreacted materials within the mixing region of interest, and it is calculated as follow (Eq. 7):

7 $$ME=\left(1-\frac{{N}_{Unmixed}}{{N}_{tot}}\right)\times 100$$

where ${N}_{Unmixed}$ represents the number of elements of the 2D grid containing the unmixed portion of the solutions with *unreacted* PLGA molecules (e.g., when the element ACN-PLGA mass fraction is greater than 0.95) and ${N}_{tot}$ is the total number of elements considered. Nevertheless, even though, according to the state-of-the-art, MI and ME are crucial variables for the assessment of the quality of the mixing of two solutions, they are not able to give any information about the process of NPs production.

To this aim, this work tries to extrapolate novel relevant data to give peculiar information in the nanosystem manufacturing process, detailing the region of the microfluidic cartridge in which the NPs are able to form, and the effect of the internal fluid dynamics onto their development and evolution. In more details combining the information provided by the velocity and mass fraction with the PLGA precipitation process, the NPs Area of Precipitation (AoP) can be identified. As described by Li et al. [24], the polymeric precipitation happens in the cartridge where the concentration of ACN is between 70 and 95%. For each numerical simulation, the ratio between the areas of cell belonging to AoP and the entire 2D surface of the serpentine microfluidic chip (Eq. 8) is calculated as follows:

8 $$AoP\%=\frac{{A}_{0.7<{X}_{ACN}<0.95}}{{A}_{tot}}\cdot 100$$

Since the NPs can be created only in the AoP, the residence time (${\tau }_{res}$) was calculated elementwise after the identification of the nanoprecipitation region. ${\tau }_{res}$ represents the time in which the NPs nucleus could grow (Eq. 9)

9 $${\tau }_{res,i}=\frac{{L}_{i}}{{v}_{i}}$$

where *i* is the element index, ${L}_{i}$ represents the distance from the outlet for the *i*^*th*^ element belonging to AoP, and ${v}_{i}$ is the velocity calculated in the centroid of the *i*^*th*^ element. In order to better explain the meaning of the variable an *ad hoc* graphical representation is present in the supplementary material.

Moreover, since the NPs required a minimum time to form (i.e., the mixing time, ${\tau }_{mix})$ calculated according to the following equation [4], (Eq. 10) the ${\tau }_{res}$ lower than the ${\tau }_{mix}$ was neglected.

10 $${\tau }_{mix}=\frac{{w}^{2}}{9\cdot D\cdot {\left(1+\frac{1}{FRR}\right)}^{2}}$$

where $w$ is the width of the channel, $D$ is the ACN to TRIS–HCl diffusion coefficient, and FRR is the flow rate ratio.

All these variables (MI, ME AoP, ${\tau }_{mix}$, and ${\tau }_{res}$) were calculated for each FRR.

### Experimental Validation

#### Set-up

**Fig. 3:**
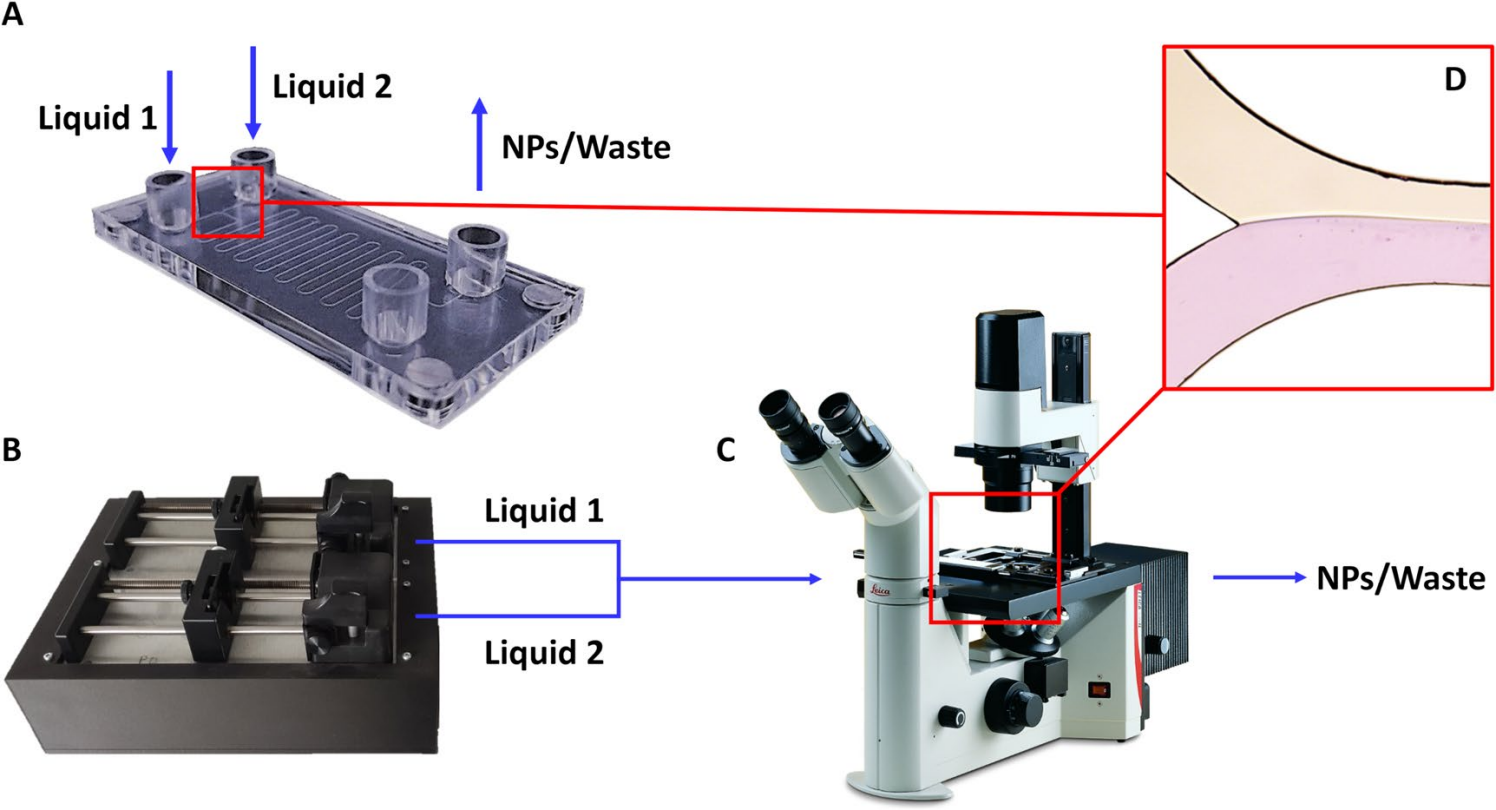
Schematic representation of the equipment used for the experimental validation. **A** Microfluidic chip adopted during the experiments; the blue arrows indicate the direction of the flow; **B** the *in-house* developed pumping system; **C** an optical microscope to observe and capture the mixing process; **D** Example of an image acquired through the microscope during the mixing process.

The experimental set-up adopted for the validation of the numerical simulations consists in three different components (Fig. 3): the Serpentine chip, a Leica DM IL LED Inverted Laboratory Microscope (Leica Microsystems, Vienna, Austria) equipped with three different magnification lenses to capture the mixing process and an *in-house* designed pumping system presented in previous works [9]. The two syringes and the microfluidic chip were connected by PTFE tubing (1/16x3/32”), while an *ad hoc* in-house Arduino-based electronic unit controlled through a mobile application was developed in order to control the produced batch volume, the TFR, and the FRR between the two pumps.

In the experiment, a TFR of 0.2 ml/min with a FRR of 1:1 was set by the pumping system to push the TRIS (dyed with rhodamine) and the organic solvent ACN-PLGA through the chip.

#### Image Acquisition and Processing

Fifteen images were acquired in the mid-point of each straight portion of the microfluidic chip with the same magnification (i.e., ×20) and without changing any parameter of the camera. The manual positioning of the microfluidic chip above the device optics was guided through the microscope on-screen real-time viewer (Alexasoft X-Entry software, Microcontrol NT, Italy)

**Fig. 4:**
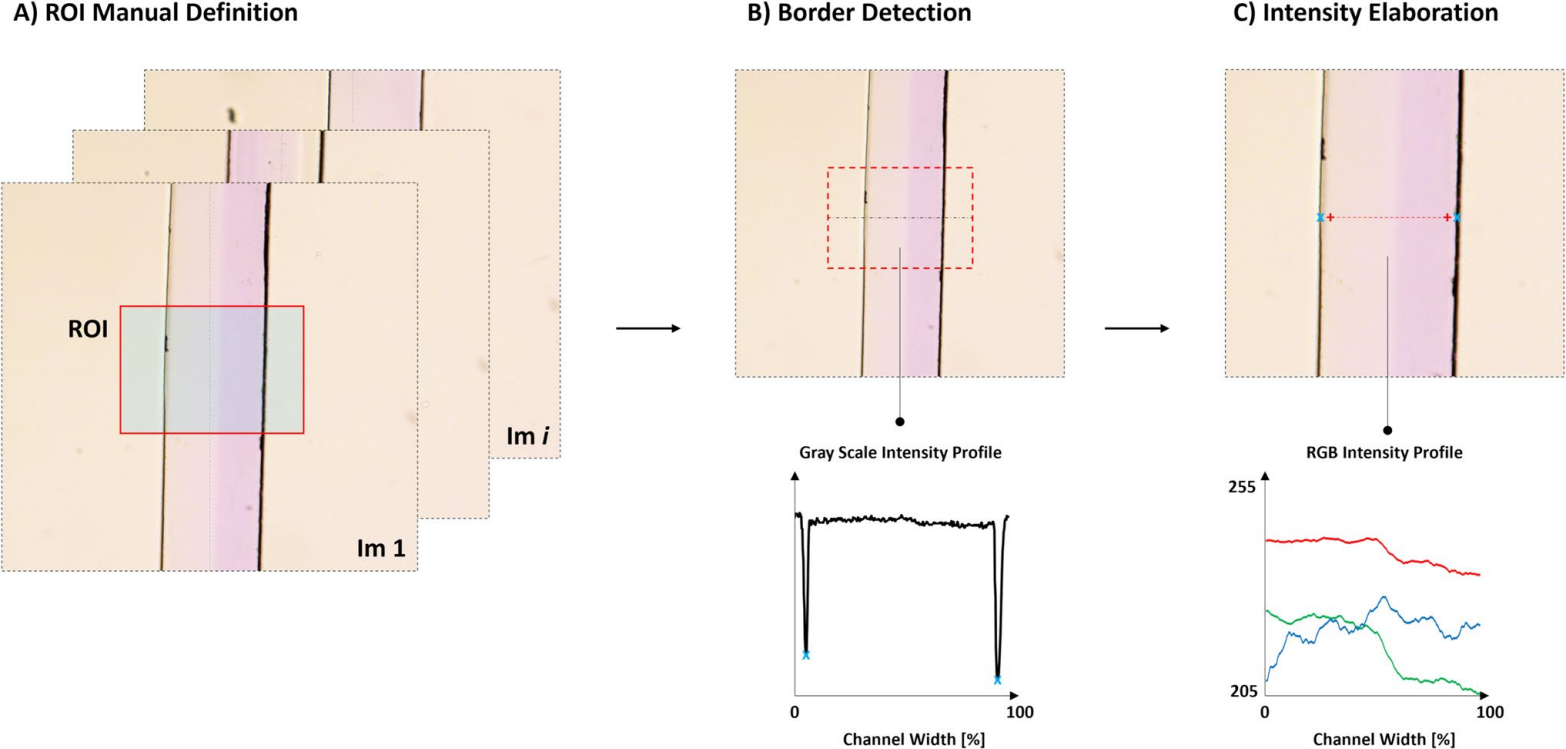
Schematic representation of the main functionality of the code realized for the image processing.

After the acquisition, the images were elaborated through an *ad hoc* semi-automatic Matlab-based software (Mathworks, Natick, Massachusetts), developed to process the data and to extract the RGB channels intensity profile (Fig. 4).

A brief description of the main functionalities of the code is reported here below:

1. Once the stack of images is imported, a rectangular region of interest (ROI) is manually drawn in the central part of the first image to cover the entire width of the channel (Fig. 4A). Moreover, since all the images were manually aligned to have the microfluidic channel centrally positioned, the first ROI can be adopted for all the images.
2. For each image, in the central portion of the ROI, the border of the channel was detected. As reported in (Fig. 4B), the border of the channel is sharply defined by two black lines. These two lines are easily detectable assessing the minimum of the gray-scale value of the image.
3. Once identified the border, the RGB intensity value of each pixel belonging to the horizontal centerline of the ROI within the channel was extracted. Furthermore, in order to be sure to neglect any shadow effect provided by the channel border, the first and the last ten pixels were not considered into the analysis (Fig. 4C).

#### Comparison with Numerical Results

To validate the results of the numerical simulation, the RGB intensity profile calculated from the experimental images were compared with the ones provided by the numerical contour plot.

For each straight portion of the microfluidic chip, the percentage difference of each RGB channel between the numerical and experimental results was evaluated as follows:

11 $${\varepsilon }_{j}=100\cdot \frac{{\overline{I} }_{Exp,ij}-{\overline{I} }_{Num,ij}}{{\overline{I} }_{Num,ij}} \hfill i=1,\dots 14 \quad j=1,..3$$

where ${\overline{I} }_{Exp,ij}$ is the mean value of the experimental image intensity for the *i*th section and *j*th RGB channel and ${\overline{I} }_{Num,ij}$ is the mean value of the numerical image intensity for the *i*th section and *j*th color.

In order to be consistent with the experimental results, the standard rainbow color bar, usually adopted to show the volume fraction contour plot in the numerical simulations, was customized using the RGB parameters of the two solutions in the unmixed state, and more details about this customization process are described in the supplementary material.

## Results

### Validation of the Numerical Model

**Fig. 5:**
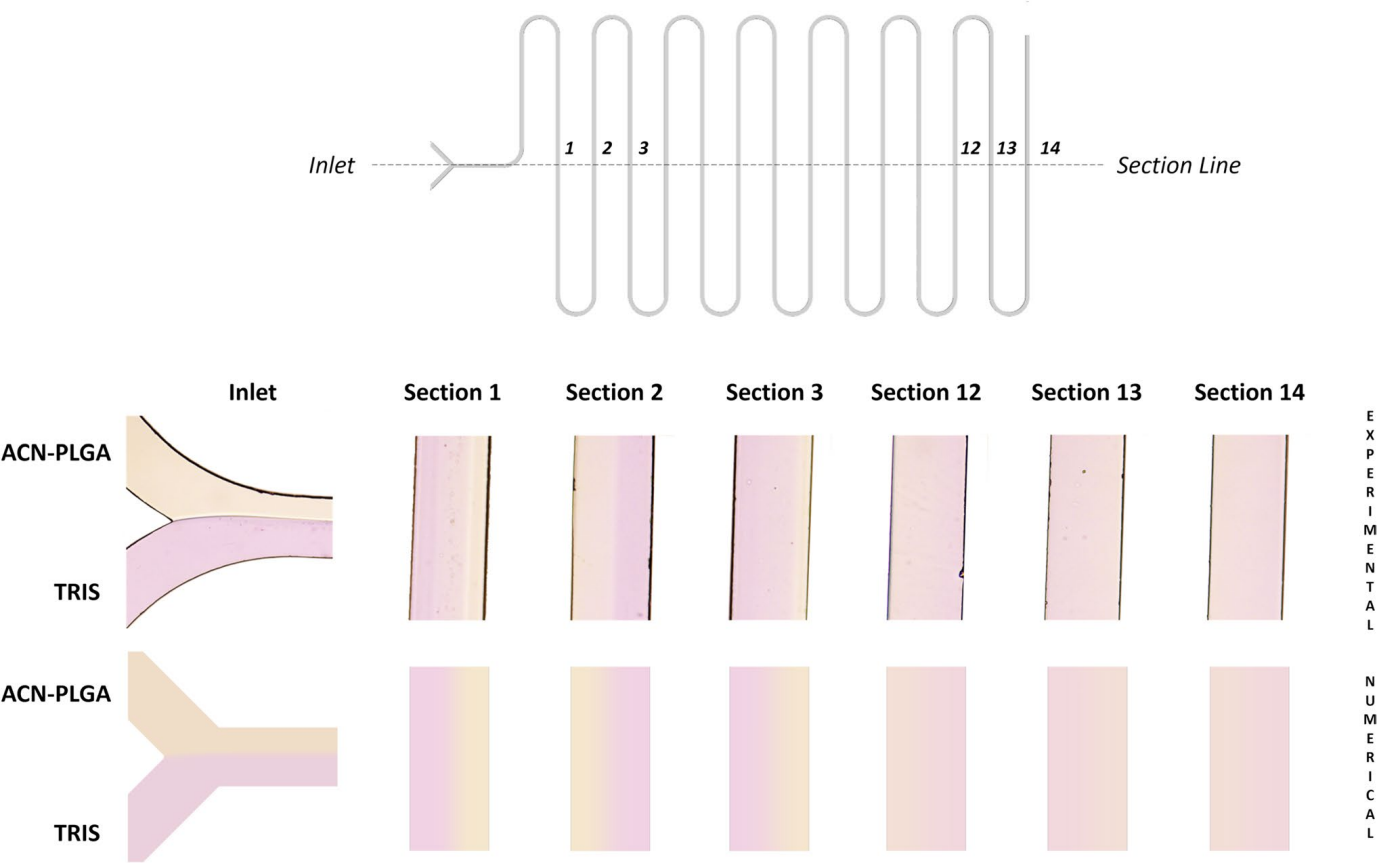
Experimental assessment (top) and numerical prediction (bottom) of the mixing process along the microfluidic chip geometry.

A preliminary qualitative comparison between numerical and experimental results is reported in Fig. 5; it is clearly visible how the two fluids begin to stir after the first bend of the chip (Section 1) and complete their mixing just before the outlet of the cartridge (Section 14), while at the inlet, thanks to the laminar flow regime, the two solutions appear to be immiscible.

**Fig. 6:**
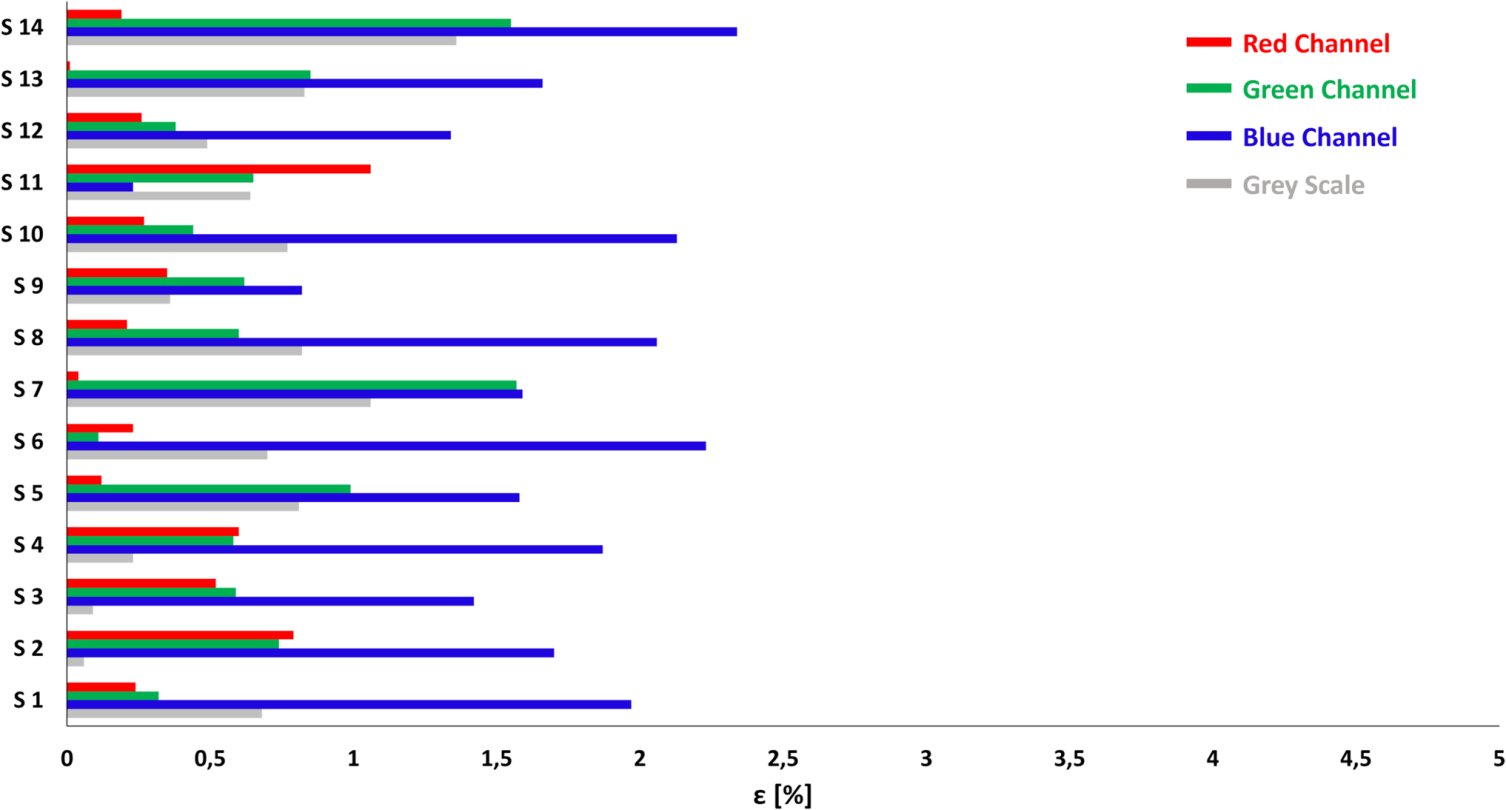
Percentage difference between the numerical and experimental approaches for the intensity of each RGB channel and in case of Grayscale.

In each section of the serpentine chip (inlet and outlet excluded), the percentage difference calculated between numerical and experimental approach is between ± 5% concerning the three RGB channels of the image and concerning the mean intensity in all the sections. This low difference confirms the agreement between the numerical representation of the phenomenon and the real mixing of the two solutions (Fig. 6).

### *Mixing Index and Mixing Efficiency*

**Fig. 7:**
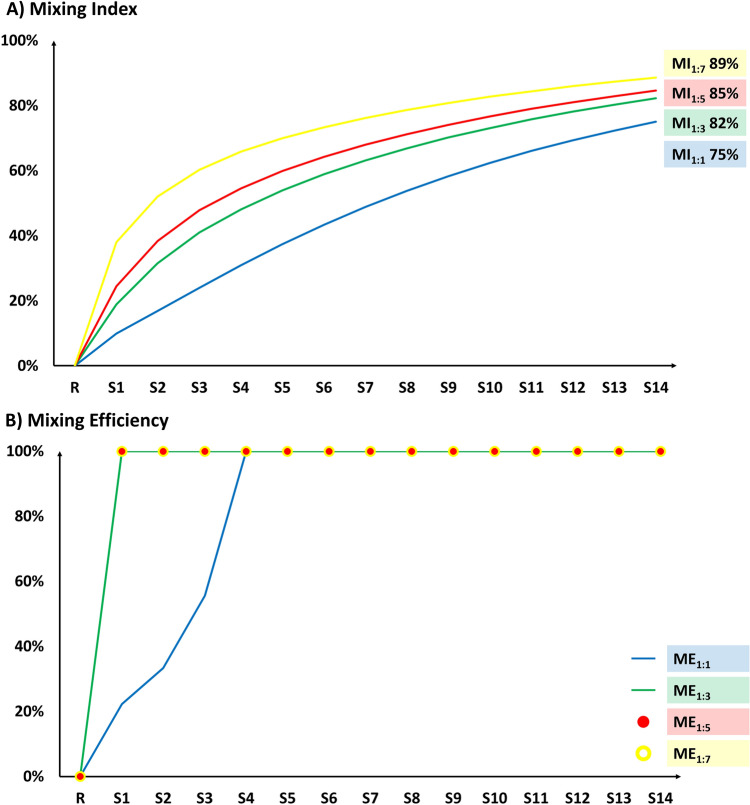
Representation of the MI (**A**) and ME (**B**) evolution along the different sections (S) of the serpentine microfluidic cartridge.

No important differences were observed about the absolute final value of MI, since it changes from the 75% in 1:1 condition to the 89% in the 1:7 condition and about its variation along the chip (Fig.7A).

Through the ME it was evident that, except the 1:1 condition, in the other fluid dynamic conditions, the two fluids can be considered completely mixed just after the first loop (Fig. 7B). Within the 1:1 condition, the PLGA polymer is able to precipitate more uniformly along the first half of the microfluidic channel.

### Novel Variables Related to NPs Production

**Fig. 8:**
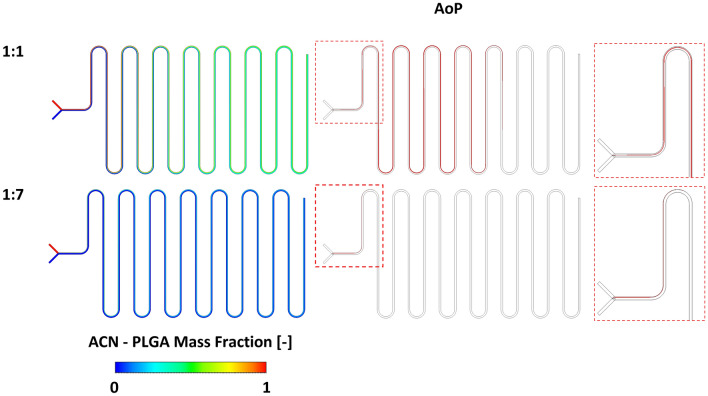
On the left, contour plots of the ACN-PLGA mass fraction distribution along the microfluidic chip with different FRR conditions (Red represents 100% ACN-PLGA, while blue represents 100% TRIS). On the right, graphical representation of the region in which NPs are forming with a magnification of the AoP in the initial part of the channel.

Beside MI and ME, no further information on the NPs formation process can be extracted by the classical analysis and therefore, we used new parameters to characterize the NPs formation. The FRR increment led to significant alteration of the ACN-PLGA distribution profiles leading to the *squeezing* of the mixing area toward the border of the channel (Fig.8). Furthermore, the FRR increase leads to a shortening of the length of the AoP. In the 1:1 condition, the AoP spread over almost the entire length of the chip, while in the 1:7 condition, the AoP is limited to the first curvature of the chip, reducing the NPs development region (Table 4).

**Table 4:** First column area of precipitation, while second and third column median and interquartile range [25th–75th percentile] of ${\tau }_{res}$ and velocity distributions calculated within the AoP at different FRR

	AoP (%)	${\tau }_{res}$(s)	*v* (m/s)
1:1	16.5	0.92 [0.78–1.25]	0.17 [0.09–0.23]
1:3	3.1	1.57 [1.23–2.12]	0.14 [0.06–0.18]
1:5	1.9	2.04 [1.45–3.74]	0.12 [0.06–0.15]
1:7	0.6	2.10 [1.92–3.81]	0.11 [0.06–0.12]

The resulting percentage of area involved in the precipitation process in relation to the total area of the channel are reported in Table 4.

The AoP decrease leads to a faster and more localized precipitation of PLGA molecules, thus inducing a more chaotic nucleation process. The increment of the FRR drive to an increment of the median ${\tau }_{res}$ and of the ${\tau }_{res}$ interquartile difference (Table 4), since the characteristic velocity of the AoP seems to decrease passing from 0.17 to 0.11 m/s (as median value).

### Manufactured NPs

From each batch, the NPs morphological characteristics were analyzed exploiting the Nicomp 380 Nanoparticle Size Analyzer (Particulate Sensors, Billerica, MA, USA). The FRR increment drives to a slight enlargement of the NPs (from 220 to 260 nm) and to a progressive reduction of the homogeneity of the product leading to a progressive increment of the PDI (Table 5). Nevertheless, it is observable that for all the working conditions, the distribution of the NPs dimension remains below 0.2 which represents an acceptable quality limit [25].

**Table 5:** Average size and PDI achieved by the produced NPs within the four different experimental conditions

	Size (nm)	PDI [–]
1:1	235.3	0.10
1:3	222.6	0.11
1:5	260.7	0.14
1:7	261.9	0.16

## Discussion

Numerical simulations represent a crucial tool for the assessment of the mixing performances of commercial or brand new microfluidic platforms adopted to produce NPs [26–28]. For the sake of simplicity, these performances are represented by two dimensionless variables: the mixing index (MI) and the mixing efficiency (ME). Nevertheless, since MI and ME are not able to deepen the NPs manufacturing, novel variables should be adopted for a comprehensive understanding of the mixing process [29].

In this context, we developed different numerical strategies able to calculate the limit working conditions of a microfluidic serpentine micromixer and to reproduce the mixing process of the two solvents. To reliably reproduce the mixing process, the rheological properties of the adopted fluids were assessed and, moreover, a dedicated image-based experimental protocol was developed and adopted to validate the *in silico* results. Finally, we have designed a novel variable able to better describe the effect of different working conditions onto the NPs precipitation process, with the aim of simplifying the formulation design, reducing the number of experiments required.

The proposed CFD model proved to be able to capture the effect of the FRR onto the mixing performances over the entire length of the chip. Moreover, it was clear that the increasing of the FRR slightly changes the final value of both MI and ME (Fig. 6A-B) evidencing the goodness of mixing in all the conditions applied without giving information about the NPs formation process. This effect is also present for the AoP, in fact just moving from 1:1 to 1:3 condition, AoP reduces from 16.5% to 3.1%. Moreover, a reduction of the mean velocity was registered in the polymer precipitation region inducing an increment of the residence time (Table 5).

To understand the efficiency of the proposed numerical model and to correlate the novel variables onto the manufacturing process, PLGA NPs were produced using the TFR calculated from the 0D model (i.e., 0.2 ml/min) and the same FRR conditions superimposed to the CFD, exploiting the machine presented in Fig. 3B. In order to have statistical significance, all the production tests were conducted in triple copy, using for each FRR condition novel chips in order to avoid any cross-contamination. No chip rupture was evinced throughout the experiment, and the NPs batch production was continuous. The numerical simulations proved to be able to properly capture the mixing of the solvent and anti-solvent basis for the NPs production; moreover, the nucleation process seems to be induced by a double phenomenon. Firstly, the increment of the FRR lead to a severe reduction of the AoP forcing the polymers to a rapid and chaotic nucleation process. Secondly, the mean velocity of the fluid within the precipitation region decreases due to the localization near the walls, increasing the residence time and worsening the aggregation process. Hence, the homogeneity of the distribution of the NPs is strongly dependent to the extent of the AoP other than the entity of the residence time.

The adoption of numerical tools proved to be important in the definition of the limit working condition of the microfluidic chip and proved to give insight into the mixing process that led to nanoparticles formation. And even though, so far, the CFD simulations are not able to give univocal thresholds, the comparative study performed in the current work may be very useful in the optimization of a DoE-based formulation process. Moving in this direction, we are currently investigating the evolution of the AoP inside a herringbone passive micromixer to observe how those structures can improve the nucleation process. At last, we are currently using the AoP to design a new microfluidic chip set for only producing NPs and confirming the universality and validity of this new parameter as a tool to optimize the production process of NPs by microfluidic precipitation.

Even though the proposed work proved to be able to capture the macroscopic mixing process induced by the geometry of the chip onto solvent and anti-solvent; due to the dimensional scale of the problem, the numerical model is not able to provide any information about the molecular diffusion of the PLGA chains from the solvent to the aqueous buffer and then, the proposed computational strategy is not able to model the NPs nucleation process.

In conclusion, this work, also through the adoption of novel variables, aims to quantitative compare the effect of different working conditions onto the macroscale fluid mixing in order to simplify the DoE strategy, trying to reduce experimental time and cost.

## Supplementary Information

Below is the link to the electronic supplementary material.

Supplementary file1 (TIF 116 kb)

Supplementary file2 (TIF 414 kb)

Supplementary file3 (TIF 1462 kb)

Supplementary file4 (TIF 660 kb)

Supplementary file5 (TIF 2267 kb)

Supplementary file6 (DOCX 4952 kb)
