# Supplementary figures and images for: Computational-Aided Approach for the Optimization of Microfluidic-Based Nanoparticles Manufacturing Process
Source: Ann Biomed Eng. 2024 Aug 4;52(12):3240–52. doi: 10.1007/s10439-024-03590-1 (PMC11561088; doi:10.1007/s10439-024-03590-1)

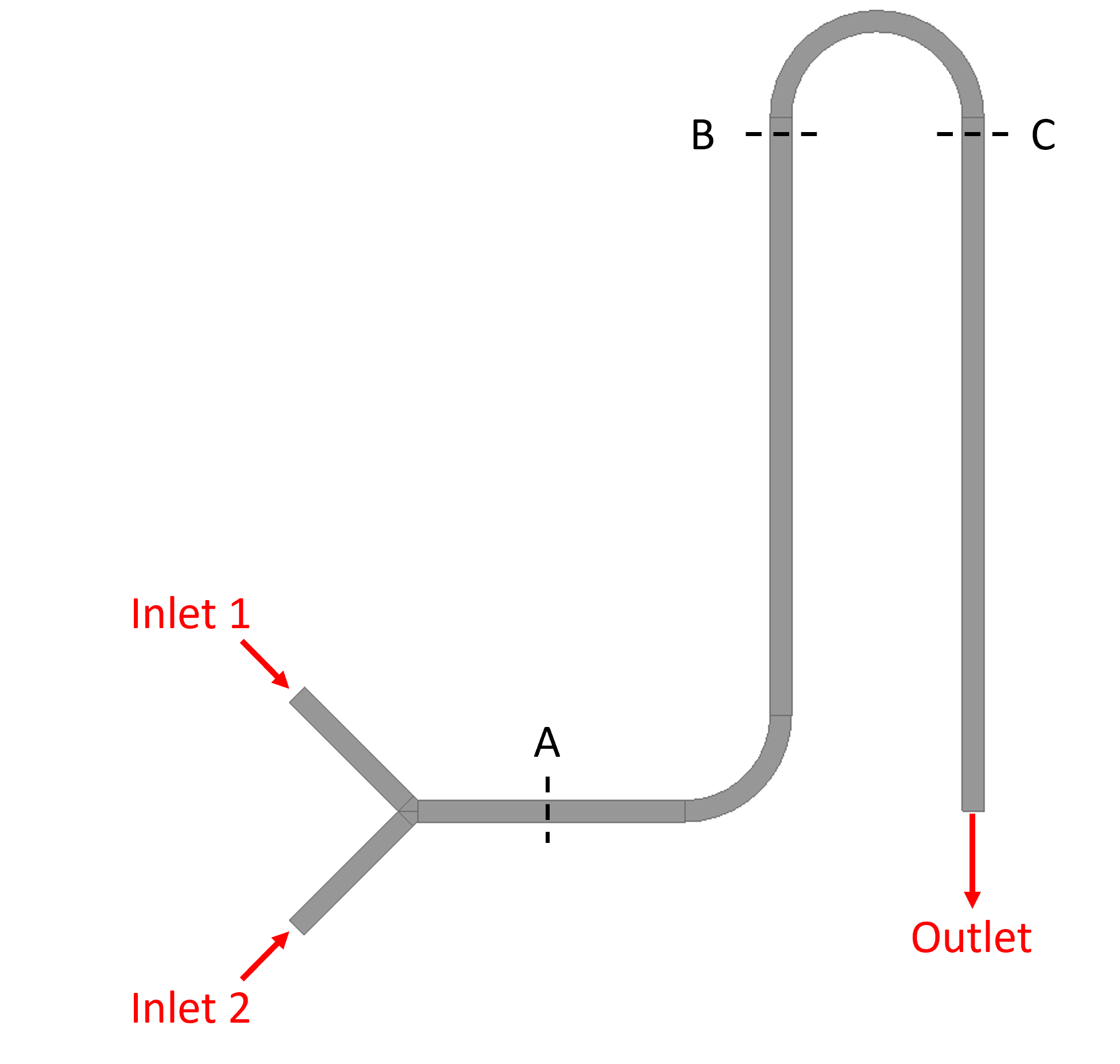

Supplement: Supplementary file 1 — Supplementary file1 (TIF 116 kb) [file 10439_2024_3590_MOESM1_ESM.tif]

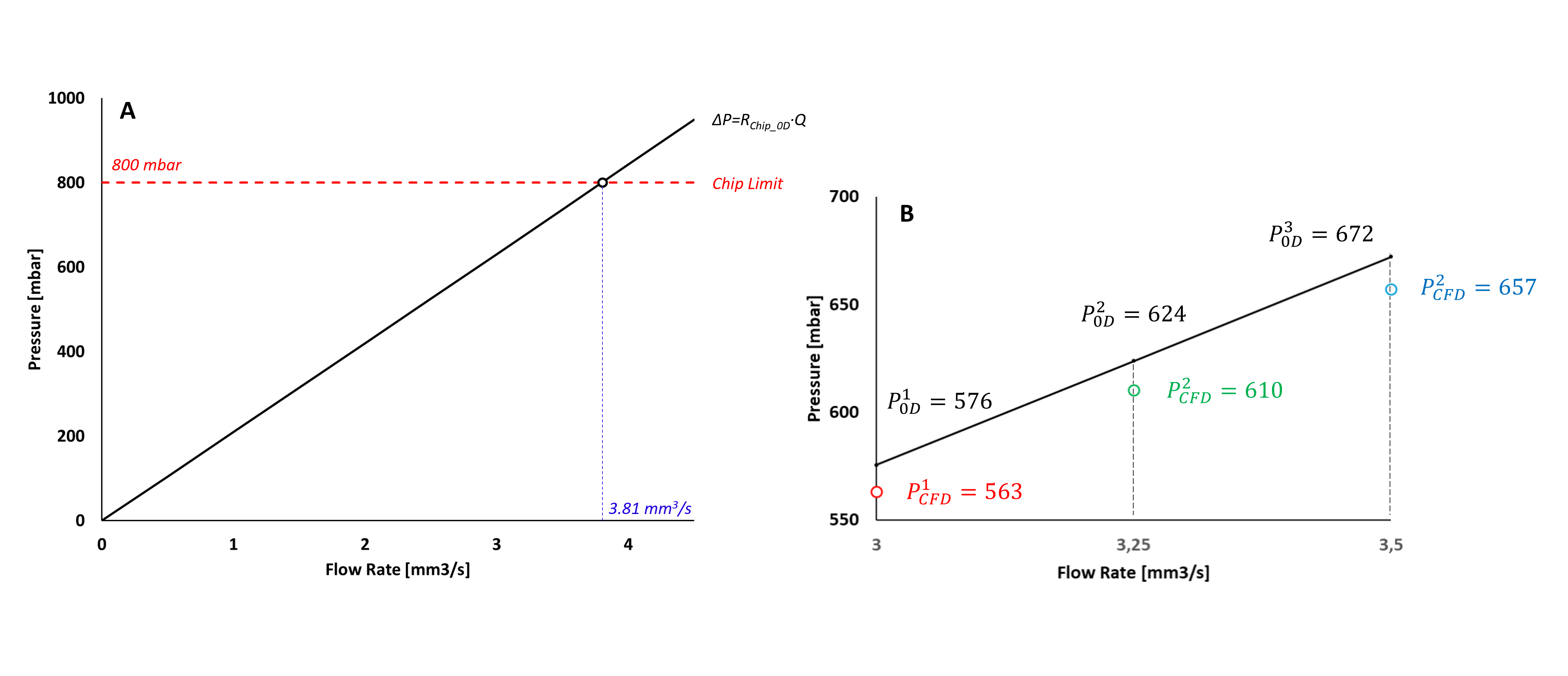

Supplement: Supplementary file 2 — Supplementary file2 (TIF 414 kb) [file 10439_2024_3590_MOESM2_ESM.tif]

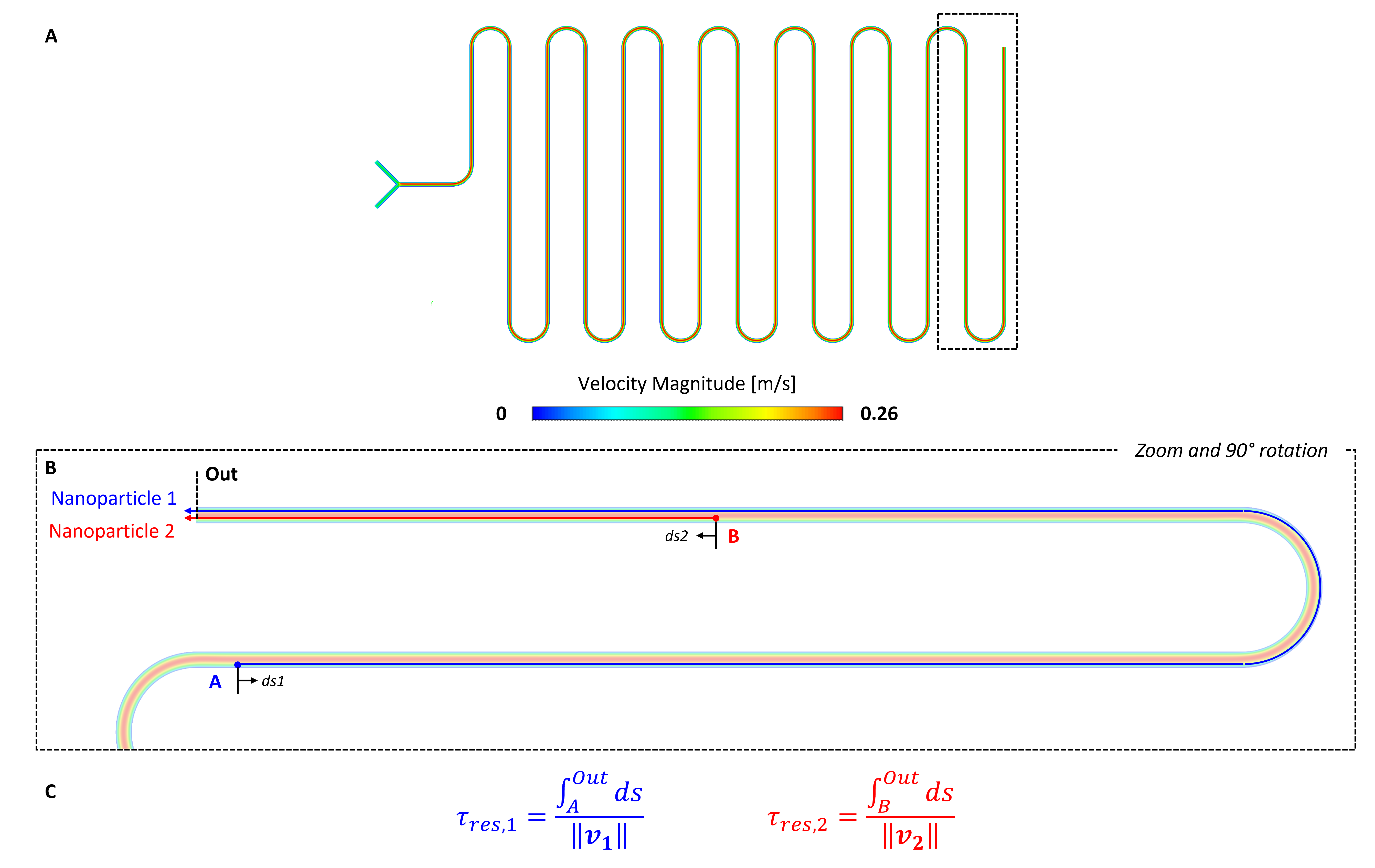

Supplement: Supplementary file 3 — Supplementary file3 (TIF 1462 kb) [file 10439_2024_3590_MOESM3_ESM.tif]

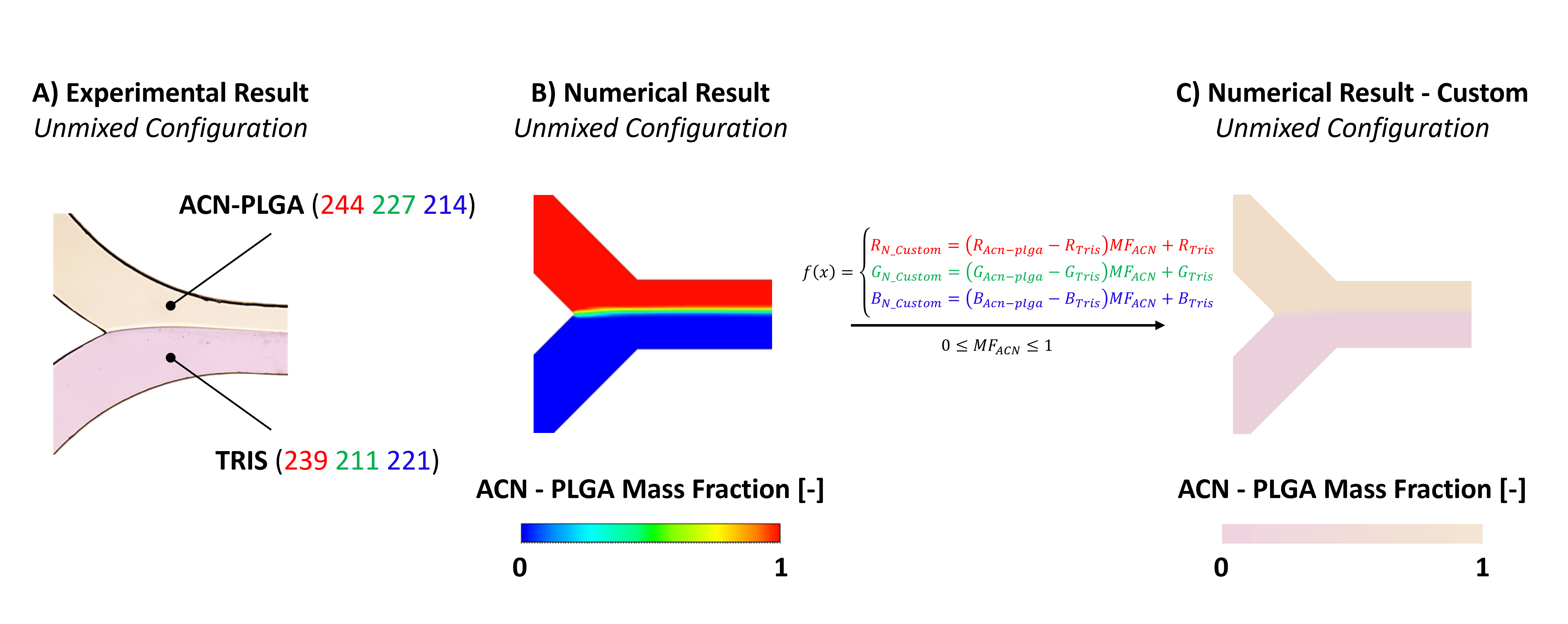

Supplement: Supplementary file 4 — Supplementary file4 (TIF 660 kb) [file 10439_2024_3590_MOESM4_ESM.tif]

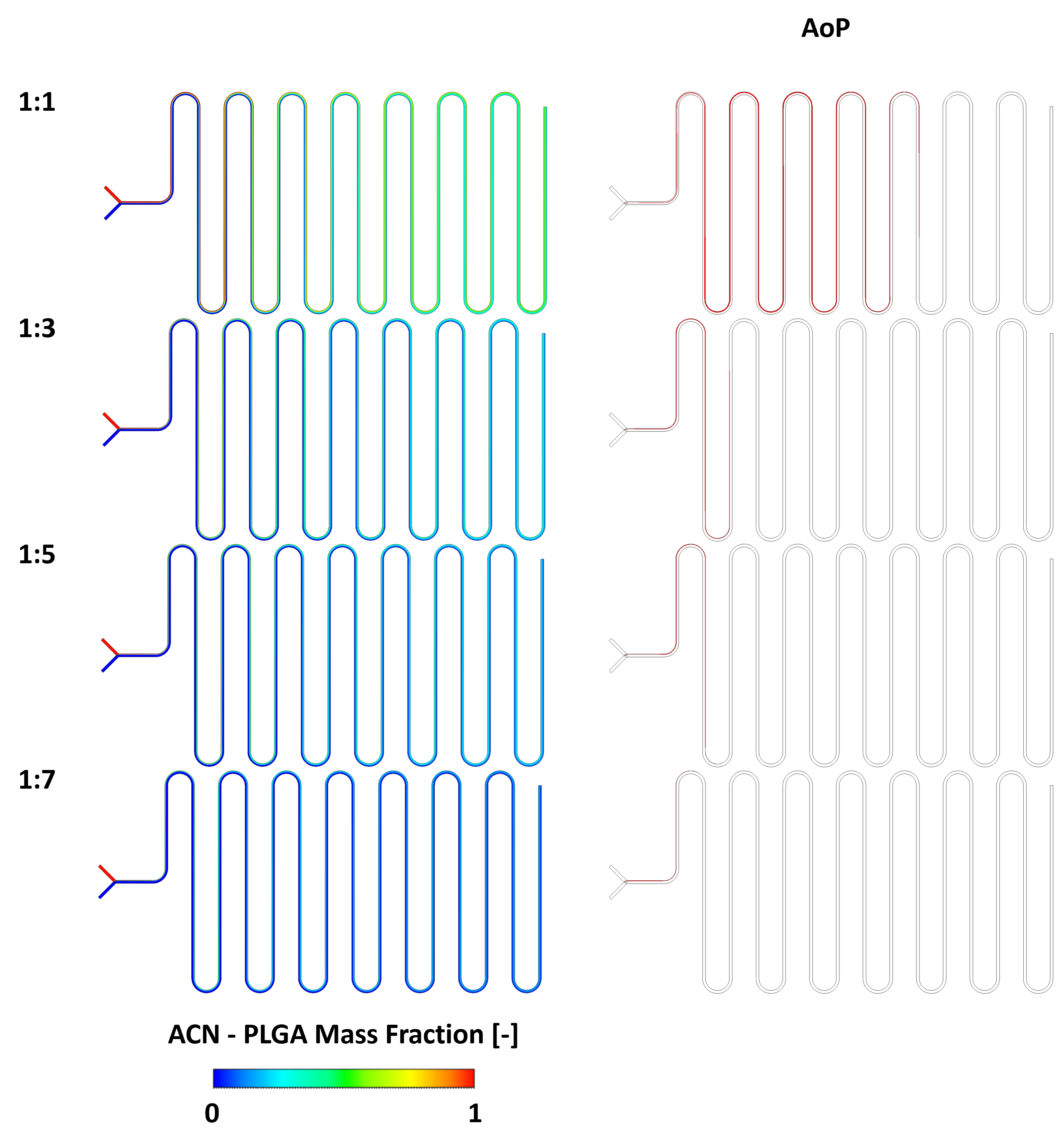

Supplement: Supplementary file 5 — Supplementary file5 (TIF 2267 kb) [file 10439_2024_3590_MOESM5_ESM.tif]
