# Supplementary material for: Computational-Aided Approach for the Optimization of Microfluidic-Based Nanoparticles Manufacturing Process
Source: Ann Biomed Eng. 2024 Aug 4;52(12):3240–52. doi: 10.1007/s10439-024-03590-1 (PMC11561088; doi:10.1007/s10439-024-03590-1)
Supplement: Supplementary file 6 — Supplementary file6 (DOCX 4952 kb) [file 10439_2024_3590_MOESM6_ESM.docx]

**SUPPLEMENTARY MATERIALS**

**Mesh Sensitivity Analysis**

The mesh independence analysis was performed between three different characteristic dimensions (*i.e., 5, 10 and 20μm*) using only part of the geometry of the entire chip (Figure S1).


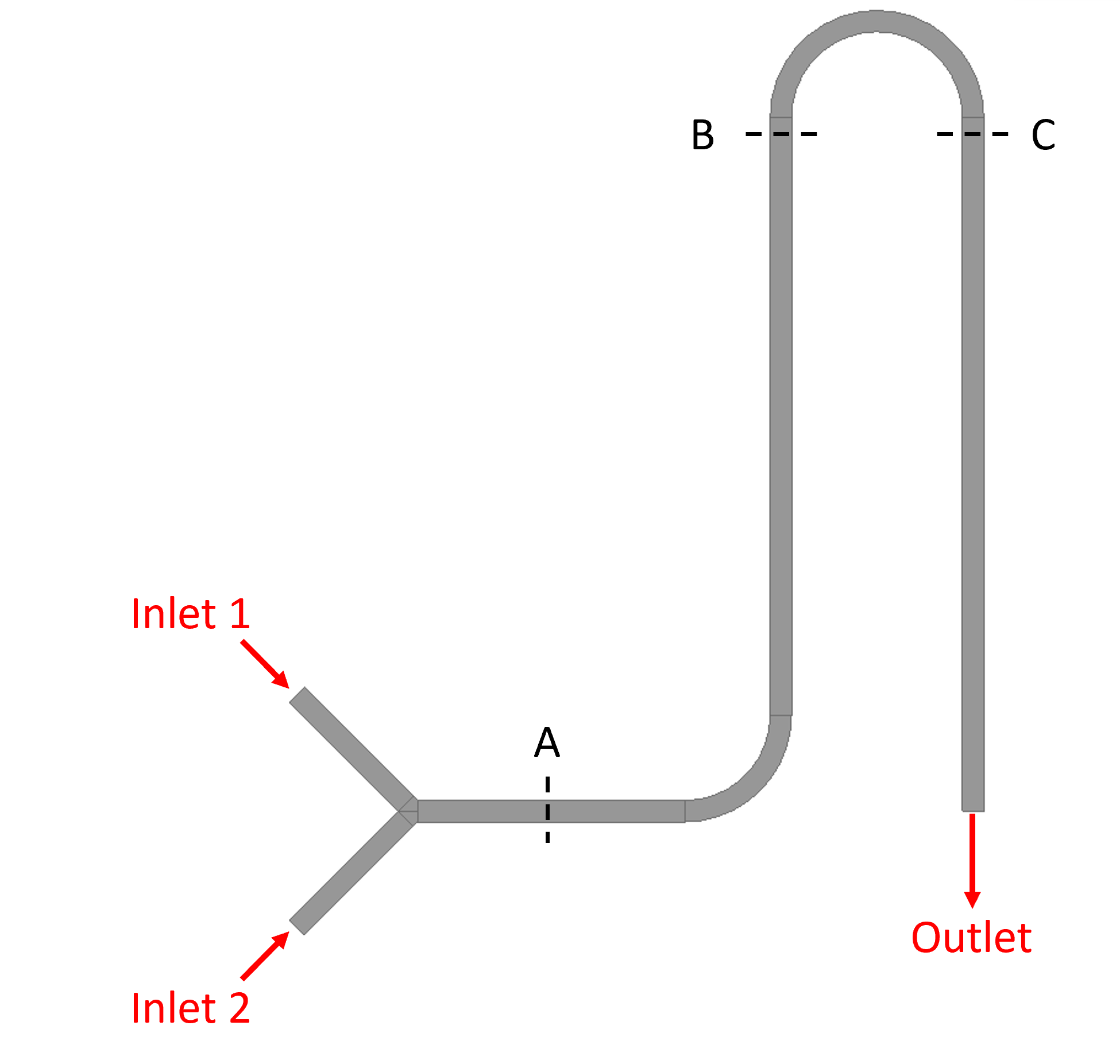


**Figure S1** Representation of the 2D geometry adopted for the mesh sensitivity analysis.

In the proposed work the results were considered independent from the adopted numerical grid when the difference between the different meshes is below 5%. All the differences are shown in Table S1.

**Table S1** Percentage error of four different variables with the three different meshes, calculated in three different point of the geometry. The grey rows of the table contain the data extrapolated at section A, the light grey ones at B, while the white ones at C.

|  | **ε_Velocity_** | **ε_pressure_** | **ε_Mass_Fraction_** | **ε_MI_** |
| --- | --- | --- | --- | --- |
| **Coarse – Medium**  **20 ÷ 10 μm** | 1.86% | 1.17% | 1.07% | - |
|  | 1.36% | 1.37% | 0.45% | 10.07% |
|  | 1.56% | 1.45% | 0.64% | 10.21% |
| **Medium – Fine**  **10 ÷ 5 μm** | 0.47% | 0.37% | 0.72% | - |
|  | 0.44% | 0.45% | 0.98% | 2.58% |
|  | 0.26% | 0.47% | 0.88% | 3.88% |

Since according to the mixing index (MI) the differences between the medium and the fine grid are largely below the 5% limit, the 10μm grid was chosen for the numerical analysis.

**Validation of the 0D model through CFD simulations**

Once identified and assessed all the components of the simplified 0D model, the total resistance of the microfluidic cartridge was calculated. This allows us to calculate the TFR limit for the rupture of the chip (*i.e.* 3.81mm^3^/s or 0.2ml/min as shown in Figure S2.A), to do so water was chosen since it is characterized by the worst rheological conditions (water viscosity is higher than ACN-PLGA viscosity).

To validate the 0D model the microfluidic cartridge was reconstructed and three different numerical simulations with a TFR of 3, 3.25 and 3.5mm^3^/s with a 1:1 FRR were performed in Ansys 2021 R2, the CFD solver settings are reported in Table S2.

**Table S2** Solver general settings.

| **Type of simulation** | Steady State |
| --- | --- |
| **Flow Regime** | Laminar |
| **Pressure-velocity scheme** | Coupled |
| **Pressure spatial discretization** | Second Order |
| **Momentum spatial discretization** | Second Order Upwind |
| **Convergence criterion** | Residual below 1e-^6^ |

As reported in Figure S2.B the difference between the pressures calculated by the 0D model and by the CFD simulations changed by 2.3% as maximum.


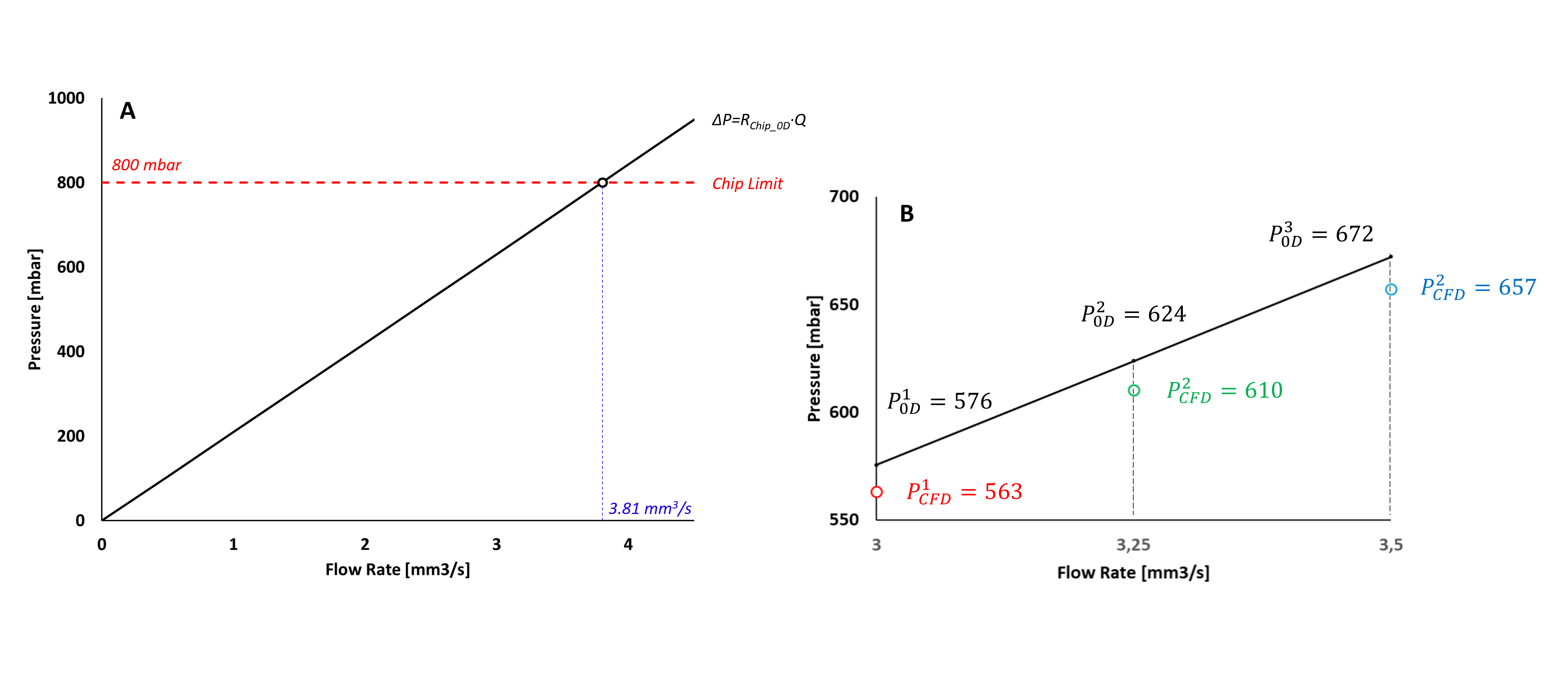


**Fig. S2** A) Graphical representation of the pressure variation, assessed through the simplified 0D model, within the chip due to the increasing of the TFR; B) Validation of the 0D model with CFD simulations at three different TFR.

Finally, since the 0D model proved to be able to predict the pressure within the chip, the maximum flowrate of 3.33mm^3^/s (*i.e.,* 0.2ml/min) was chosen as a boundary condition for both the CFD simulations and the manufacturing process. This value allows to simplify the device settings, which adopts ml/min for the flow measurement, remaining below the chip pressure limit.

**Derivation of the characteristic equations of the numerical model**

The aim of this paragraph is to show the characteristic equations of an incompressible and Newtonian fluid within a region of interest considering the problem by an eulerian point of view.

**Conservation of Mass – Continuity Equation**

|  | $\frac{\partial\rho}{\partial t}+\nabla\cdot\left( \rho\mathbf{v} \right)=S_{m}$ | (1) |
| --- | --- | --- |

In Eq. 1 $\rho$ is the density of the fluid, $\mathbf{v}$ is the velocity of the stream and $S_{m}$ is a source term. Since the flow is incompressible (i.e., $\frac{\partial\rho}{\partial t}=0$) and since there is no source mass (i.e., $S_{m}$=0) Eq 1 can be simplify into:

|  | $\nabla\cdot\left( \rho\mathbf{v} \right)=0$ | (2) |
| --- | --- | --- |

Showing that all the fluid mass is pass through the control volume without any accumulation or depletion.

**Conservation of momentum – Definition of Navier Stokes equation**

The principle of conservation of the momentum is formulated starting from the Newton’s second law

|  | $m\mathbf{a=}\sum\mathbf{F}_{\mathbf{ext}}$ | (3) |
| --- | --- | --- |

If we consider the effect of the fluid viscosity, of the gravity and of any external volumetric force, Eq.3 can be expressed as the following equation:

|  | $\rho\frac{\partial\left( \mathbf{v} \right)}{\partial t}+\rho\left( \mathbf{v}\cdot\nabla\right)\mathbf{v}=\nabla\cdot\boldsymbol{\sigma}+\rho\mathbf{g}+\mathbf{F}$ | (4) |
| --- | --- | --- |

Where, $\rho\mathbf{g}$ is the gravitational force, $\mathbf{F}$ are volumetric external forces and $\boldsymbol{\sigma}$ represent the stress tensor that can be expressed as a function of the fluid viscosity and of the hydrostatic pressure (Eq. 5):

|  | $\boldsymbol{\sigma}=\mu\left( \nabla\mathbf{v}+\nabla\mathbf{v}^{T} \right)-p\mathbf{I}$ | (5) |
| --- | --- | --- |

If we calculate the derivatives of the stress tensor, Eq. 4 turns into Eq. 6:

|  | $\rho\frac{\partial\left( \mathbf{v} \right)}{\partial t}+\rho\left( \mathbf{v}\cdot\nabla\right)\mathbf{v}=-\nabla p+\mu\left( \nabla^{2}\mathbf{v} \right)+\rho\mathbf{g}+\mathbf{F}$ | (6) |
| --- | --- | --- |

Finally, thanks to the assumptions adopted in our model, many components of the equation can be neglected. $\frac{\partial\left( \rho\mathbf{v} \right)}{\partial t}=0$ because of the steady state hypothesis, moreover$\rho\left( \mathbf{v}\cdot\nabla\right)\mathbf{v}$=0 since the flow is strongly laminar and $\rho\mathbf{g}=0$ and $\mathbf{F}=0$ because in microfluidics gravity and volumetric force can be neglected.

Hence, the momentum equation can be simplified in Eq. 7:

|  | $\nabla p=\mu\left( \nabla^{2}\mathbf{v} \right)$ | (7) |
| --- | --- | --- |

Showing the direct dependencies between the pressure change, the velocity and the viscosity.

**Conservation of Species - Species Transport Equation**

The species transport equation is adopted to solve the mass conservation of different chemical species ($Y_{i}$) within a control volume (Eq.6):

|  | $\frac{\partial\left( \rho Y_{i} \right)}{\partial t}+\nabla\cdot\left( \rho\vec{v}Y_{i} \right)=-\nabla\cdot\mathbf{J}_{\mathbf{i}}$ | (8) |
| --- | --- | --- |

Due to the steady state assumption, $\frac{\partial\left( \rho Yi \right)}{\partial t}=0$, the transport equation can be simplified as:

|  | $\nabla\cdot\left( \rho\mathbf{v}Y_{i} \right)+\nabla\cdot\mathbf{J}_{\mathbf{i}}=0$ | (9) |
| --- | --- | --- |

Furthermore, the diffusion flux $\mathbf{J}_{\mathbf{i}}$ can be calculated, adopting the Fick’s Law for laminar flow, as reported in Eq. 10:

|  | $\mathbf{J}_{\mathbf{i}}=-\rho D_{i,m}\nabla Y_{i}-D_{T,i}\frac{\nabla T}{T}$ | (10) |
| --- | --- | --- |

In which $D_{i,m}$ represents the mass diffusion coefficient and $D_{T,i}$ is the thermal diffusion coefficient. In our case the problem is isothermal and therefore the diffusion flux can be simplified in this way:

|  | $\mathbf{J}_{\mathbf{i}}=-\rho D_{i,m}\nabla Y_{i}$ | (11) |
| --- | --- | --- |

**Calculation of** $\boldsymbol{\tau}_{\boldsymbol{res}\boldsymbol{,}\boldsymbol{i}}$

A schematic process involved in the nanoparticle $\tau_{res,i}$calculation is herein displayed to better understand and visualize the meaning of the variable


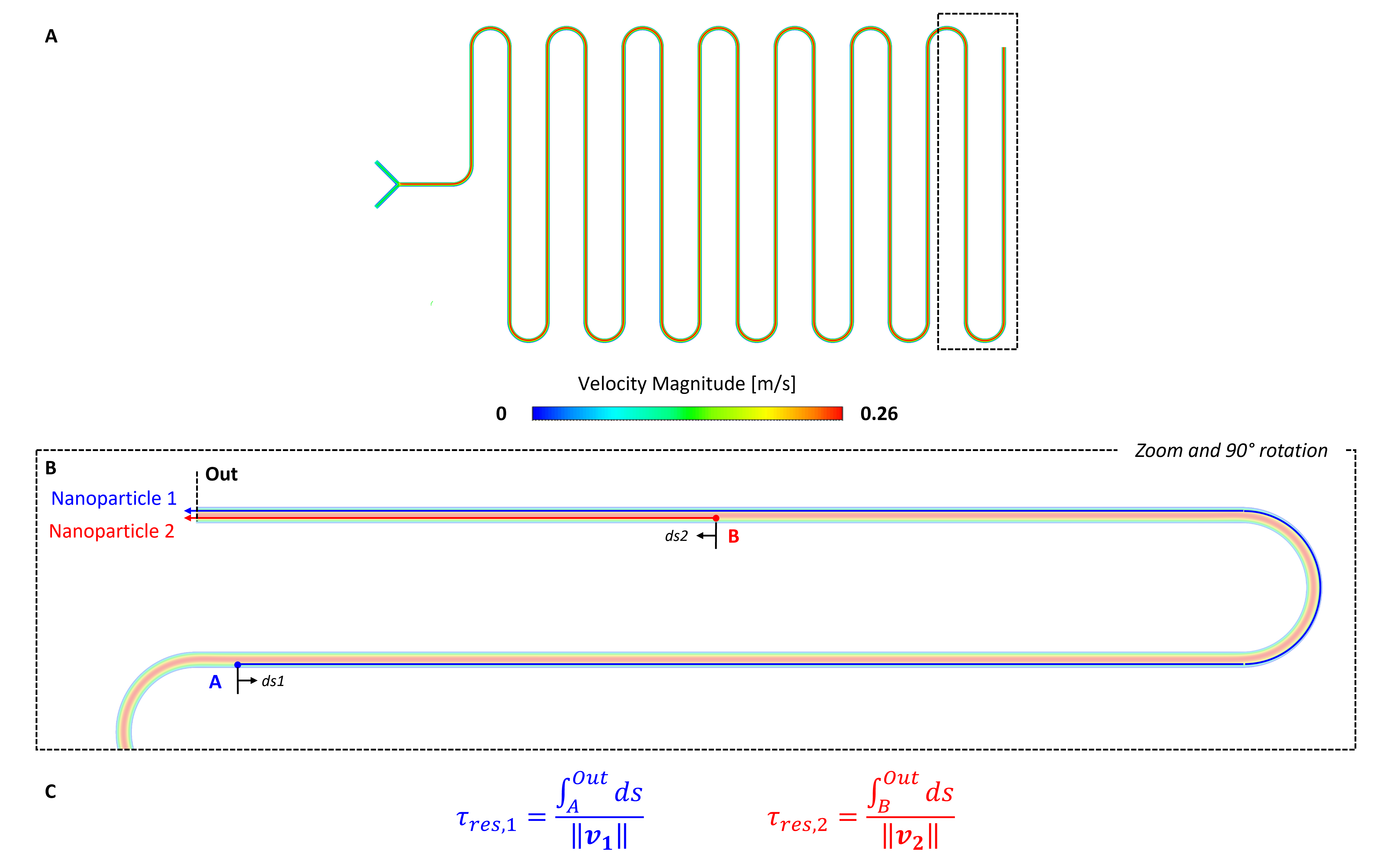


**Fig. S3** A) Contour plot of the velocity profile in the microfluidic domain with a TFR=0.2ml/min and a 1:1 FRR; B) Magnification of the outlet portion of the chip reporting potential trajectories of nanoparticles; C) Mathematical formulation of $\tau_{res,i}$; the line integral at the numerator represents the length of the trajectory experienced by the *i^th^* nanoparticle, while the denominator represents the velocity magnitude.

**Colorbar customization for the validation of the numerical simulation**

To customize the numerical mass fraction contour plot, the RGB data of each unmixed fluid was extracted from the experimental image acquired at the inlet junction of the serpentine microfluidic chip (Figure S4.A). Moreover, since the fluid mass fraction (MF) is a normalized variable (*i.e.,* 0≤MF≤1) Figure S4.B, the colour transformation was performed applying to the numerical results the function *f(x)* elementwise (Figure S4.C).

**
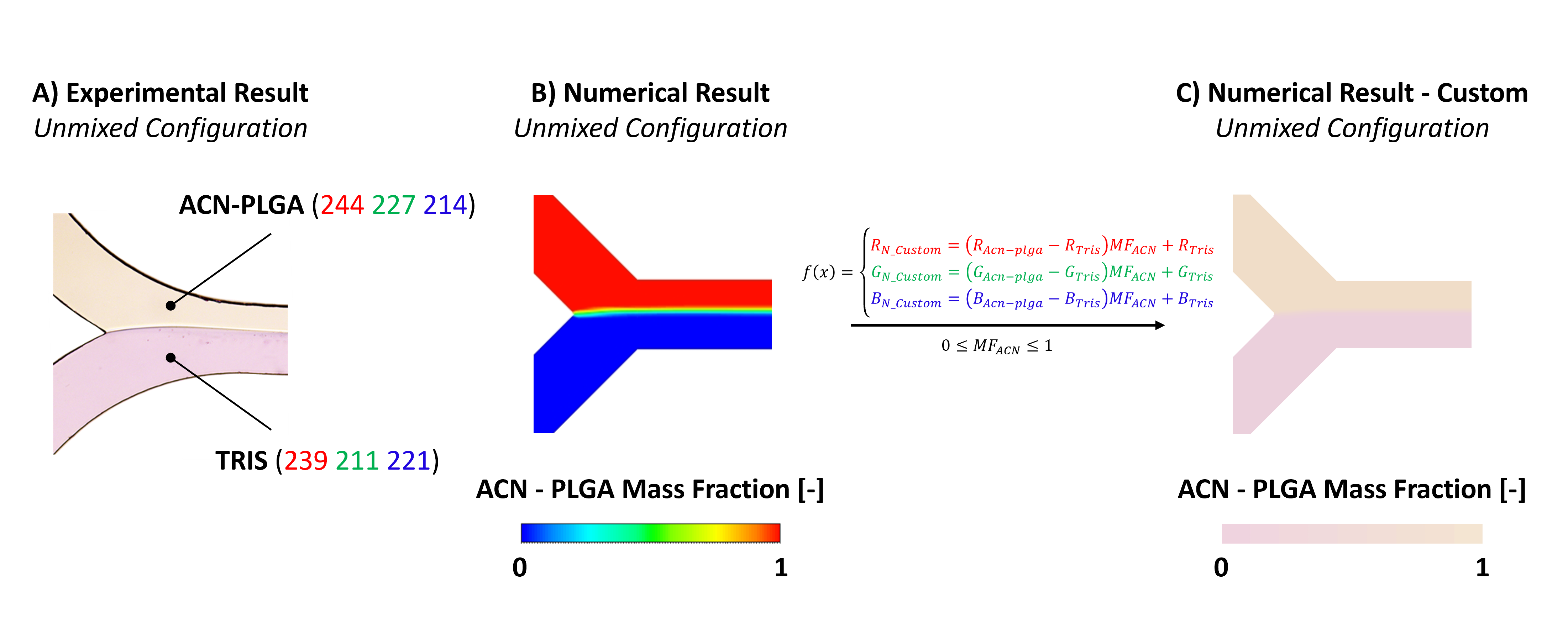
**

**Fig. S4** A) Image of the experimental result at the Y junction with the information about the RGB parameter of each unmixed fluid; B) Standard rainbow colormap of the distribution of ACN mass fraction; C) Customized version of the numerical results obtained elementwise through the transformation function *f(x). MF_ACN_*: ACN mass fraction; *R_N_Custom_*, *G_N_Custom_*. *B_N_Custom_*: Red, Green and Blue value of the numerical contour-plot after customization; *R_Acn-plga_*, *G_Acn-plga_*, *B_Acn-plga_*: Red, Green and Blue value of the unmixed ACN solution; *R_Tris_*, *G_Tris_*, *B_Tris_*: Red, Green and Blue value of the TRIS buffer.

**Numerical Results**

A comprehensive visualization of the results of the numerical simulations is reported in this section. All the contour maps derived from different boundary conditions show the impact of the FRR on both the mixing profile and the area of precipitation within the microfluidic channel.


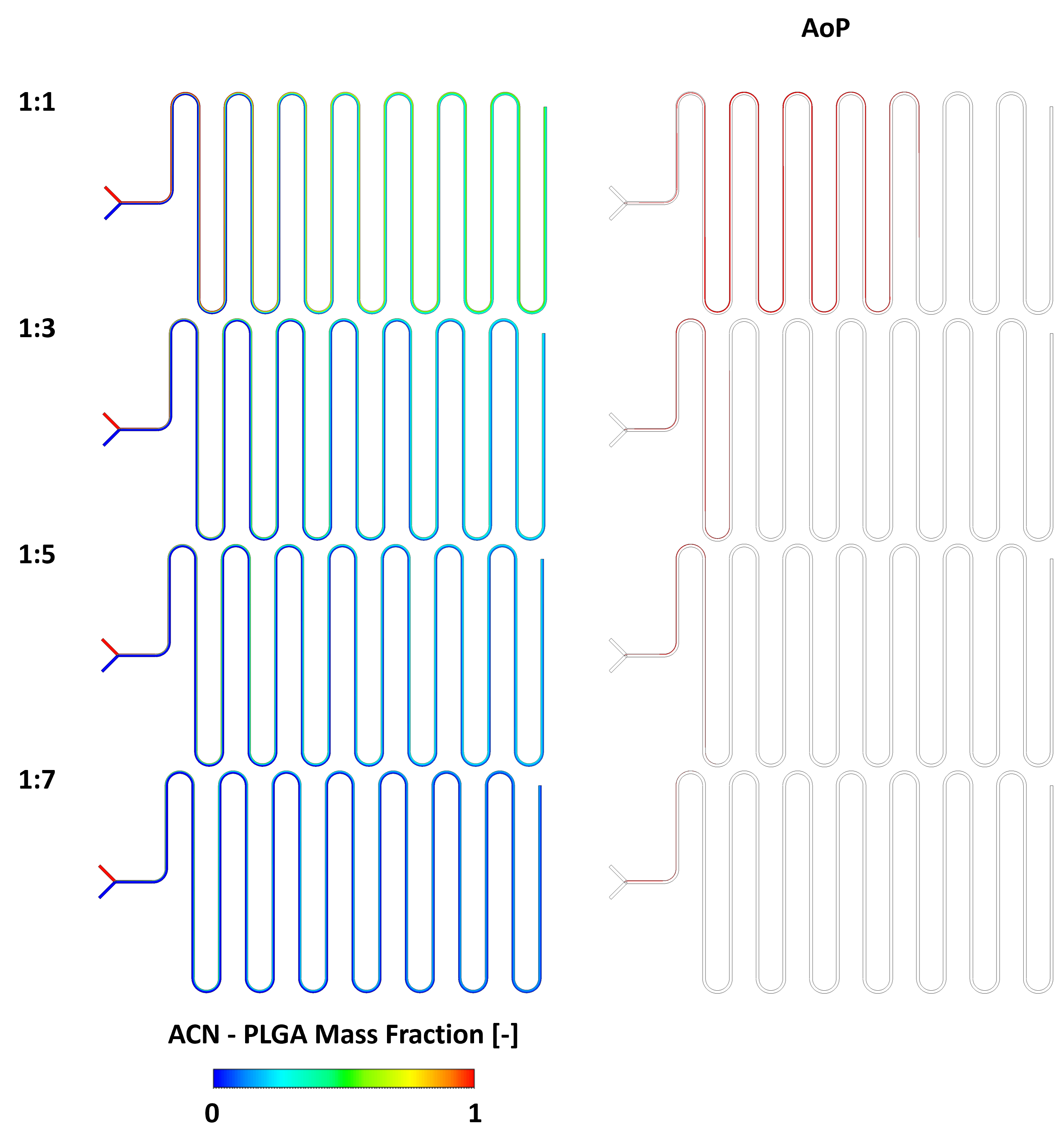


**Fig. S5** On the left, contour plots of the ACN-PLGA mass fraction distribution along the microfluidic chip with different FRR conditions (Red represents 100% ACN-PLGA while blue represents 100% TRIS). On the right, graphical representation of the region in which NPs are forming
